# Diagnostic psychiatric and somatic comorbidity in patients with depression in the Western Balkan countries

**DOI:** 10.1371/journal.pone.0295754

**Published:** 2024-01-02

**Authors:** Milan Latas, Branko Stefanovski, Alma Mihaljević-Peleš, Amra Memić Serdarević, Izet Pajević, Nera Zivlak Radulović, Sabina Radulović, Bojana Đukić, Vasilije Korugić, Željko Jovandić

**Affiliations:** 1 Faculty of Medicine and University Clinical Centre of Serbia, University of Belgrade, Belgrade, Serbia; 2 Ss. Cyril and Methodius University, Skopje, North Macedonia; 3 University Hospital Centre Zagreb, Zagreb, Croatia; 4 Clinical Center University of Sarajevo, Sarajevo, Bosnia and Hercegovina; 5 University Clinical Center Tuzla, Tuzla, Bosnia and Hercegovina; 6 University Clinical Centre of the Republic of Srpska, Banja Luka, Bosnia and Hercegovina; 7 Psychiatric Hospital of Canton Sarajevo, Sarajevo, Bosnia and Hercegovina; 8 Institute for Medical Research, University of Belgrade, Belgrade, Serbia; 9 Health Center “Dr Simo Milošević”, Belgrade, Serbia; 10 Special Hospital for Psychiatric Diseases "Kovin", Kovin, Serbia; PLOS: Public Library of Science, UNITED KINGDOM

## Abstract

**Introduction:**

This paper aims to examine the frequency and significance of diagnostic comorbidity of psychiatric disorders and somatic diseases in a sample of patients with depression as well as present current psychopharmacological treatment of the patients in the sample.

**Methods:**

The subjects in this study sample were 489 patients from the four Western Balkan countries with current primary diagnosis of major depression according to ICD 10. Comorbid psychiatric disorders and non-psychiatric illnesses were noted according to ICD 10 criteria during the diagnostic interview and analysed later. Additionally, the pharmacological treatment (existing and newly introduced) for each patient was noted and analysed later.

**Results:**

At least one comorbid psychiatric disorder was present in 72.5% of patients. The most frequent were anxiety disorders (53.6%), specifically generalized anxiety disorder (20.2%); non-organic sleep disorders (50.7%), specifically insomnia (48.4%); and sexual dysfunctions (21.4%), specifically lack of sexual desire (20.2%). Comorbidity with any non-psychiatric illness was present in 80.3% of patients. The most frequent were circulatory system diseases (55.9%), specifically hypertension (45.9%); endocrine, nutritional and metabolic disorders (51.3%), specifically hyperlipidaemia (24.0%); and other non-psychiatric disorders (60.7%), specifically low back pain (22.7%). All patients received pharmacological treatment with different medications. Most patients received monotherapy or combination therapy of antidepressants, anxiolytics, antipsychotics and antiepileptics. The most frequently used antidepressants were escitalopram, sertraline, and duloxetine. The most frequently used anxiolytics were alprazolam and diazepam, the most used antiepileptic was pregabalin, and the most used antipsychotics were olanzapine, quetiapine, and aripiprazole.

**Conclusion:**

The results of the study confirm the results of previous research studies about the high prevalence of psychiatric and non-psychiatric comorbidities in patients with depression that were conducted in the past. It would be important if future studies could prove the importance of those comorbidities on clinical severity, choice of treatment, and its outcome in patients with depression.

## Introduction

Comorbidity is one of the concepts in psychiatry that arose as a consequence of the modern diagnostic system [1]. This means that previous diagnostic systems contained a significantly smaller number of diagnostic categories and were significantly more broadly conceived. Current diagnostic systems have a significantly larger number of diagnostic categories, and there is a significantly greater possibility of analysing comorbid disorders. This concept allowed psychiatrists to look at their patients from multiple aspects, which ensured that important information in the patient’s clinical severity were not neglected. The existence of clear diagnostic criteria and no hierarchical rule for establishing a diagnosis have given researchers the opportunity to analyse many aspects of complex psychiatric disorders. Clinicians often use this concept in formulating recommendations for therapy and seeking a pragmatic approach to possible complications of some psychiatric disorders [2]. During the last decades, the concept of comorbidity has taken hold in psychiatry to a significant extent [3].

Although the original concept of comorbidity refers to any medical illness that is present in one patient, it has become usual in psychiatry to examine only psychiatric syndromes under this term [3]. So, for example, it is common to examine only the presence of other psychiatric disorders under the term comorbidity of depression [4]. Studies examining the frequency of psychiatric comorbidity in subjects with depression indicate that 74% of subjects with depression met the diagnostic criteria for at least one other psychiatric disorder, and that as many as a third of subjects with depression met the criteria for three or more disorders [4]. At the same time, the most frequent comorbidities for the depressive disorder were anxiety disorders (58%) and disorders due to abuse of alcohol and other substances (42%). Among anxiety disorders, the most common were social phobia (27%), specific phobia (24%), post-traumatic stress disorder (19%), and generalized anxiety disorder (17%). Among substance use disorders, alcohol abuse or addiction was the most common (29%) [5]. Other psychiatric disorders were significantly less common: dysthymia 9%, somatoform disorders 23%, impulse disorders 19%, and eating disorders 4% [4]. However, for a considerable period of time there has been a lack of quality studies on depression comorbidity with larger sample sizes relying on routine clinical practice. Thus, it remains unclear whether there have been any changes over time in the frequency and significance of comorbidity of depression.

On the other hand, research studies have shown that depression does not occur only in comorbidity with other psychiatric disorders, but that it can occur simultaneously with many somatic diseases. The results of research studies indicate that depression in some somatic diseases occurs significantly more often than in the general population. Thus, for example, it was indicated that depression occurs in 40–65% of patients with myocardial infarction, in 25% of patients with cancer, in 25% of patients with cerebrovascular diseases, and with significantly more frequent occurrence in patients with multiple sclerosis, rheumatoid arthritis and psoriasis relative to the general population [3, 6]. Nevertheless, all of these studies were conducted on samples of patients with primary somatic illnesses and resulted in a lack of research study results regarding the frequency of comorbid somatic illness in a sample of patients with depression.

Therefore, this paper aims to examine the frequency and significance of diagnostic comorbidity of psychiatric disorders and various somatic diseases in a sample of patients with depression, and to present current psychopharmacological treatment of the patients in the sample. The research hypothesis is that there is a high frequency of diagnostic comorbidity of psychiatric disorders and somatic diseases in patients with depression. This hypothesis was formed on the basis of clinical experience and on the basis of the cited research studies that examined comorbidity of depression.

## Method

This study is part of a larger research project known as the ‘COSMOS study,’ which aims to identify the incidence of comorbidities and therapeutic procedures in selected neurological and psychiatric diagnoses. The study seeks to evaluate the differences in therapy between patients with comorbidities and those without them, like the study from Dragašek (2019) [7]. The COSMOS study focused on several diagnostic groups as the primary diagnoses under investigation: Parkinson’s disease, Alzheimer’s disease, depression, anxiety, schizophrenia, bipolar affective disorder, and neuropathic pain. Each monitored area had its own parameters of investigation.

A primary diagnosis refers to the disorder that is most distressing to the patient and is the primary reason for the patient to seek help from a specialist in psychiatry or neurology. The COSMOS study was a prospective, naturalistic, longitudinal, non-intervention, observational multi-centre study conducted in over 174 clinician practices at 46 different clinical treatment facilities in four countries in the Western Balkans region: Serbia (18 centres), Croatia (5 centres), Bosnia and Herzegovina (4 centres), and North Macedonia (19 centres). There were 96 investigators from Serbia, 10 investigators from Croatia, 22 investigators from Bosnia and Herzegovina, and 46 investigators from North Macedonia. Only investigators with appropriate specialization, completed required education for conducting the study, and at whose site the agreement for study participation was signed were included in the study as study investigators.

The COSMOS study included adult participants (> 18 years old) diagnosed with Parkinson’s disease, Alzheimer’s disease, depression, anxiety disorder, schizophrenia, bipolar affective disorder, and/or neuropathic pain, who voluntarily participated in the study, were able to follow the study protocol, and had signed a written consent form. This article presents the findings regarding patients whose primary diagnosis was depression. The recruitment period for this study was from 1^st^ December 2019 to 1^st^ December 2021.

The study was reviewed and approved by independent ethics committees and/or regulatory authorities in participating countries in line with the local legislation requirements for epidemiological studies. In Bosnia and Herzegovina: Ethical Committee of the Clinical Center University of Sarajevo number 03-02-50626, of 7 October 2019; Agency for medicinal products and medical devices, number: 08–07.5-1-9963-1/19 of 18 October 2019. In Serbia: Ethical Committee of the University Clinical Centre of Serbia in Belgrade, number 274/13, of 29 November 2019; Ethical Committee of Instutute od Mental Health, number 2057/1, of 17 December 2019. In Croatia: Ethical Committee of the University Clinical Centre Zagreb, Class: 8.1-19/260-2, Number: 02/21 AG, of 25 November 2019; Ethics Committee of the Zagreb-West Health Centre, of 20 December 2019; Ethics Committee of the Primorsko-Goranska County Health Centre, No.: 01-7/7-6-20 of 23 January 2020. In North Macedonia: Commission for Clinical Trials of Drugs and Medical Devices and National Ethics Committee, number 11-6812/3, of 2 September 2019.

Patient’s written consent was the first procedure carried out during the study baseline visit. The information was explained verbally by the investigator in a manner understandable to the patient. Only patients who gave their voluntary consent to participate in the study (Informed Consent Form, ICF), were further included in the study. Furthermore, data was collected only from patients who gave their consent for the collection, analysis and reporting of their personal data in compliance with Regulation (EU) 2016/679 on the protection of natural persons with regard to the processing of personal data (General Data Protection Regulation, GDPR).

### Subjects

The subjects in this study sample were 489 patients with different clinical types of depression according to ICD 10. That means that depression was the main reason for a patient to seek treatment from a psychiatrist, neurologist or neuropsychiatrist. Patients with primary diagnoses of any other psychiatric disorders were excluded from the study sample as they did not align with the study objectives. There were 39.5% (n = 193) patients from Serbia, 22.7% (n = 111) from Bosnia and Herzegovina, 22.7% (n = 111) from North Macedonia, and 15.1% (n = 74) from Croatia. Table 1 presents basic demographic and clinical data of the sample.

**Table 1:** Demographic data.

Demographic data*	
**Mean age**	52.3 ± 13.0 (range 19–85) years
**Gender**	
• **Male**	65.4% (n = 320)
• **Female**	32.5% (n = 159)
**Marital status**	
• **In a relationship or married**	66.5% (n = 325),
• **Single**	13.3% (n = 65)
• **Divorced**	11.0% (n = 54)
• **Widowed**	8.8% (n = 43)
**Employment**	
• **full-time**	40.7% (n = 199)
• **unemployed**	26.6% (n = 130)
• **retired**	23.7% (n = 116)
• **part-time**	8.6% (n = 42)
**Included by**	
• **psychiatrists**	74.0% (n = 362)
• **neuropsychiatrists**	8.8% (n = 43)
• **neurologists**	6.7% (n = 33)

*No data were available on: gender for 10 (2%) patients, on marital status for 2 (0.4%), on employment status for 2 (0.4%), and who included for 51 (10.4%) patients.

Among 489 patients, 65.4% (n = 320) were diagnosed with a depressive episode, 29.7% (n = 145) were diagnosed with recurrent depressive disorder (relapse), 2.7% (n = 13) with dysthymia–a chronic depression of mood, 3.1% (n = 15) with organic depression disorder, 1.6% (n = 8) with recurrent brief depressive episodes, and 1.0% with atypical depression.

In the subgroup of patients with depressive episodes, the prevalence of the different types of episodes were as follows: 13.4% (n = 43) of patients had mild depression, 47.8% (n = 153) patients had moderate depression, 32.5% (n = 104) of patients had severe depression without psychotic symptoms, and 6.3% (n = 20) of patients had severe depression with psychotic symptoms. In the subgroup of patients with recurrent depressive disorder, the prevalence was as follows: 8.3% (n = 12) of patients had mild depression, 40.0% (n = 58) of patients had moderate depression, 36.6% (n = 53) of patients had severe depression without psychotic symptoms, and 15.2% (n = 22) of patients had severe depression with psychotic symptoms. On average, the patients included in the sample (n = 455) had an average of 2.2 ± 1.5 of depressive episodes within the last 2 years.

### Procedure

The study sample consisted of patients who sought psychiatric treatment at various psychiatric facilities across four countries. These patients were referred by primary care physicians and other psychiatrists, or sought treatment on their own accord for mental health issues. The patients included in the study samples underwent a semi-structured interview with a specialist of psychiatry to assess the presence of psychiatric disorders and somatic illnesses related to their mental and somatic health problems. Patients diagnosed with depression were included in the study sample after their diagnosis was established according to ICD 10 criteria and after obtaining their informed consent in accordance with the aims of the study. The comorbid psychiatric disorders and non-psychiatric illnesses according to ICD 10 criteria were recorded during the diagnostic interview and analysed afterwards. Additionally, the pharmacological treatment for each patient, both existing and newly introduced, was recorded and analysed later. All patients were seen by their physician who also participated as an investigator in the study.

### Statistical analyses

Summary statistics include the number of patients/observations, frequencies and corresponding percentages for categorical variables. For continuous variables, descriptive statistics (number of patients/observations, mean, median, standard deviation, minimum and maximum, first and third quartile) have been tabulated. All analyses in this statistical report were performed only for patients with available data. Due to the aims of the study, the effects of therapy and thorough statistical analysis and data correlations were not analysed. Microsoft Office Excel 2016© was used for the computational part of the analysis, and Microsoft Office Word 2016© was used to compile the report and prepare the paper.

## Results

### Psychiatric comorbidity of depression

Among patients with recorded data, at least one comorbid psychiatric disorder was present in 72.5% of patients. On average, patients (n = 484) had 1.2 ± 1.1 comorbid psychiatric disorder diagnoses. 27.5% (n = 133) of patients diagnosed with depression had no comorbid psychiatric disorder diagnoses, 38.0% (n = 184) had one comorbid psychiatric disorder diagnoses, 20.9% (n = 101) had two comorbid psychiatric disorder diagnoses, 11.0% (n = 53) had three, 2.3% (n = 11) had four, and 0.4% (n = 2) had five or more comorbid psychiatric disorder diagnoses.

Table 2 displays the frequency of psychiatric comorbidities within the sample, with each patient possibly having several comorbid diagnoses. Most frequent were anxiety disorders, specifically generalized anxiety disorder; non-organic sleep disorder, specifically insomnia; and sexual dysfunction, specifically lack of sexual desire.

**Table 2:** Frequency of psychiatric comorbidities (n = 351).

Psychiatric comorbidities	n (%)
**Anxiety disorders**	**188 (53.6%)**
→Generalized anxiety disorder	71 (20.2%)
→Panic disorder	27 (7.7%)
→Post-traumatic stress disorder	27 (7.7%)
→Obsessive-compulsive disorder	6 (1.7%)
→Other anxiety disorders	65 (18.5%)
**Sleep disorder (non-organic)**	**178 (50.7%)**
→Insomnia	170 (48.4%)
→Hypersomnia	8 (2.3%)
**Sexual dysfunction**	**75 (21.4%)**
→Lack of sexual desire	71 (20.2%)
→Excessive sexual drive	4 (1.1%)
**Disorder due to alcohol or other psychoactive substance use**	**53 (15.1%)**
**Eating disorder**	**32 (9.1%)**
→Anorexia or bulimia	16 (4.6%)
→Overeating	16 (4.6%)
**Somatoform disorder**	**15 (4.3%)**
**Psychotic disorder**	**9 (2.6%)**
→Schizoaffective disorder	3 (0.9%)
→Schizophrenia	2 (0.6%)
→Other psychotic disorder	4 (1.1%)
**Bipolar affective disorder**	**5 (1.4%)**
**Dementia**	**5 (1.4%)**
**ADHD**	**0 (0.0%)**
**Other psychiatric disorder not listed above**	**39 (11.1%)**

Each patient could possibly have several comorbid diagnoses.

### Non-psychiatric comorbidity of depression

Comorbidity with any non-psychiatric illness was present in 392 (80.3%) out of 489 patients. Each patient could possibly have several comorbidities. The average number of comorbid non-psychiatric disorder diagnoses per patient was 1.4 ± 1.0 (n = 489). Out of the total sample of 489 patients, 96 (19.7%) did not have any comorbid non-psychiatric illness diagnosis. However, 173 (35.5%) patients had one comorbid non-psychiatric disorder, 132 (27.0%) had two, 80 (16.4%) had three, 6 (1.2%) had four, and 1 (0.2%) patient had five or more comorbid non-psychiatric illnesses diagnoses.

Table 3 displays the frequency of non-psychiatric comorbidities in the sample, with each patient possibly having several comorbid diagnoses. Most frequent were diseases of the circulatory system, specifically hypertension; endocrine, nutritional and metabolic disorders, specifically hyperlipidaemia; and other non-psychiatric disorders, specifically low back pain.

**Table 3:** Frequency of non-psychiatric comorbidities in the sample (n = 392).

Non-psychiatric comorbidities	n (%)
**Diseases of the circulatory system**	**219 (55.9%)**
→Hypertension	180 (45.9%)
→Arrhythmia	23 (5.9%)
→Stroke	22 (5.6%)
→Coronary artery disease	20 (5.1%)
→Peripheral vascular disease	14 (3.6%)
→Heart failure	12 (3.1%)
→Orthostatic hypotension	10 (2.6%)
**Endocrine, nutritional and metabolic disorders**	**201 (51.3%)**
→Hyperlipidaemia	94 (24.0%)
→Diabetes mellitus	59 (15.1%)
→Obesity	53 (13.5%)
→Disorder of thyroid gland	49 (12.5%)
→Metabolic syndrome	10 (2.6%)
→Hyperprolactinemia	2 (0.5%)
**Diseases of the nervous system**	**35 (8.9%)**
→Polyneuropathy	18 (4.6%)
→Parkinsonism	10 (2.6%)
→Epilepsy	9 (2.3%)
**Diseases of the respiratory system (asthma, COPD or other)**	**13 (3.3%)**
**Other non-psychiatric disorders**	**238 (60.7%)**
→Low back pain	89 (22.7%)
→Headache	80 (20.4%)
→Pain in limb	58 (14.8%)
→Myalgia (muscle pain)	42 (10.7%)
→Stomach pain	41 (10.5%)
→Palpitations	18 (4.6%)
→Neoplasms	18 (4.6%)
→Renal disease	14 (3.6%)
→Liver disease	12 (3.1%)
→Haematological disorders	12 (3.1%)
→Infectious diseases	4 (1.0%)
→Other non-psychiatric disorder not listed above	55 (14.0%)

Each patient could possibly have several comorbid conditions.

### Pharmacological treatment

Every patient (100%) in the sample received pharmacological treatment with different medicines. Table 4 displays the distribution of patients who were treated with a specific group of medicines. The majority of patients in the sample were treated with antidepressants, anxiolytics, antipsychotics, and/or antiepileptics, either as monotherapy or in combination with each other, or in combination with other listed groups of medicines.

**Table 4:** Distribution of patients who were treated with a specific group of medicines whose frequency was ≥1% (n = 489).

Pharmacological treatment	n	%
Antidepressant	479	98.0%
Anxiolytic	295	60.3%
Antipsychotic	170	34.8%
Antiepileptic	120	24.5%
Hypnotic and sedative	60	12.3%
Analgesic	23	4.7%
ACE inhibitor in monotherapy and combination	12	2.5%
Antidementive	10	2.0%
Antiparkinsonian drug	8	1.6%
Beta-blocker	8	1.6%
Thyroid drug	7	1.4%
Drug used in addictive disorder	6	1.2%
Drug for acid related disorder	5	1.0%

Each patient could possibly have several groups of medicines included in their ongoing therapy.

Table 5 displays the average total daily dose and distribution of patients treated with a specific medicine (INN). The most frequently used antidepressants were escitalopram, sertraline, duloxetine and venlafaxine. The most frequently used anxiolytics were alprazolam and diazepam, the most frequently used antiepileptic was pregabalin, and the most frequently used antipsychotics were olanzapine, quetiapine, and aripiprazole.

**Table 5:** Average total daily dose and distribution of patients treated with a specific medicine (INN).

Patients with recorded ongoing pharmacological treatment (n = 489)	n	%	Average total daily dose (in appropriate units)
Escitalopram	146	29.9%	12.4
Sertraline	142	29.0%	74.2
Alprazolam	122	24.9%	1.0
Diazepam	79	16.2%	10.8
Duloxetine	50	10.2%	55.8
Bromazepam	49	10.0%	6.0
Olanzapine	48	9.8%	6.3
Venlafaxine	48	9.8%	123.8
Clonazepam	46	9.4%	2.5
Mirtazapine	46	9.4%	24.6
Quetiapine	42	8.6%	128.3
Pregabalin	39	8.0%	130.4
Zolpidem	38	7.8%	8.8
Lorazepam	37	7.6%	3.2
Paroxetine	37	7.6%	25.3
Aripiprazole	34	7.0%	11.5
Trazodone	30	6.1%	115.8
Lamotrigine	30	6.1%	105.0

Each patient could possibly have several medications included in their ongoing therapy.

## Discussion

The co-occurrence of two or more psychiatric disorders at the same time in the same person and the co-occurrence of psychiatric disorders and somatic illnesses is a complex phenomenon that can have an impact on various aspects of these disorders and illnesses. For example, individuals with depression and comorbid conditions may experience a more severe and prolonged course of illness compared to those with depression alone. With this research, we attempted to bridge a significant time gap in previous studies on this topic. This time gap may be viewed as a barrier to understanding mental health problems in everyday clinical practice. The problem with psychiatric comorbidity is that some diseases are often overlooked and neglected, which can have significant and negative impacts on patients, their treatments, and their quality of life.

Before discussing them, the results of this study should be interpreted with caution. In this study, we collected information about the patients’ medical illnesses through interviews, but a complete medical examination was not conducted. This limitation prevents us from concluding that all the relevant data was collected and included in the results. Additionally, assessing personal information about the mental and medical status of patients with depression can be challenging as their current mood may affect the accuracy of their reports on functioning and other pertinent data. Also, in this research, a categorical approach was applied, but in future research it would be important to apply a dimensional approach with the inclusion of instruments for the assessment of psychopathological phenomena.

### Psychiatric comorbidity of depression

The findings of our study revealed that over half of the patients with primary depression had comorbidities with other psychiatric conditions or somatic illnesses. The most prevalent comorbid condition was in the spectrum of anxiety disorders, which is consistent with previously published studies [4, 8, 9]. For example, according to Fava et al., 2000, comorbid anxiety disorder diagnoses were present in 51% of consecutive depressed outpatients between the ages of 18 and 65 years [8]. In an epidemiological study done by Kessler et al., 2005, 62% of individuals with major depressive disorder also met the criteria for generalized anxiety disorder, 52% for social phobia, 50% for posttraumatic stress disorder, 48% for panic disorder, and 42% for obsessive-compulsive disorder [9]. All of these results are expected considering that symptoms of depression and anxiety frequently co-occur, regardless of whether depression or an anxiety disorder is considered as the primary disorder.

Our study revealed that non-organic sleep disorder and sexual dysfunction were next in line as the most common comorbid conditions in patients with depression. Our findings related to the prevalence of insomnia are consistent with the results of other clinical studies. According to Winokur A et al., 2001, insomnia may occur in 60–80% of patients with major depression [10]. Given that non-organic sleep disturbances, in particular insomnia, are among the criteria for diagnosing depression, these findings did not come as a surprise to us. Furthermore, this raises the question of whether this is a true comorbidity of psychiatric conditions or simply an overlapping diagnostic criterion for various psychiatric disorders categorized as distinct categories in diagnostic manuals.

On the other hand, the findings of our study indicate a lower prevalence of sexual dysfunction compared to other clinical studies. According to Thakurdesai & Sawant, 2018, sexual dysfunctions were seen in 63% of patients with mild to moderate stage of depression [11]. This discrepancy could be due to a different methodology and different study sample. But the issue of frequent comorbidity of sexual dysfunction and depression could be viewed as a consequence of overlapping diagnostic criterions. Moreover, patients who experience sexual dysfunction most frequently report a lack of desire, which has an impact on their relationships and quality of life. This further complicates the healing process because it increases the patients’ sense of loneliness. This could potentially be a symptom of depression, a consequence of the use of antidepressants, or it could be an independent disorder diagnosed as a comorbid condition.

### Non-psychiatric comorbidity of depression

Furthermore, our study revealed a high prevalence of comorbid somatic illnesses in patients diagnosed with depression. The most common diagnosed comorbid conditions were diseases of the circulatory system, mostly hypertension. In addition, endocrine, nutritional, and metabolic disorders, as well as other conditions such as low back pain and headache, were also found to be among the most frequent non-psychiatric conditions in patients with depression. Our data shows that diseases of the cardiovascular system had the highest number of reported comorbid conditions, consistent with the data obtained in previous studies. However, the order of comorbidities is somewhat different (Robertson & Katona, 1997; Kupfer & Frank, 2003) [3, 6]. Additionally, it is worth noting that another study (Scalco et al., 2005) found evidence supporting increased prevalence of hypertension in depressed patients, increased prevalence of depression in hypertensive patients, as well as an association between depressive symptoms and hypotension, and alteration of the circadian variation of blood pressure in depressed patients [12]. In general, all of these results and previous reports suggest that depression is associated with the presence of cardiovascular problems and diseases [13].

On the other hand, our findings show that, rather than cancer as a comorbid condition, endocrine, nutritional, and metabolic issues ranked second after circulatory diseases in the list of most prevalent comorbidities. Given the average age of the subjects, who were over 50 years old, and the high prevalence of these diseases in the general population of this age group, it is not surprising that our study identified these as the most common non-psychiatric comorbid conditions in patients with depression.

One of the most frequently present non-psychiatric comorbidities in our study were pain syndromes such as low back pain, pain in limb, myalgia, headache, and stomach pain. In general, pain syndromes frequently co-occur in depression. According to the findings of other clinical studies, the reported prevalence of pain in depressed patients varies from 50% to 60% of individuals with depression [14, 15]. Additionally, some clinical studies have shown that physical symptoms, including pain, are the primary motive for patients to seek medical attention [15]. As pain has a significant impact on both treatment outcomes and the functioning of patients with depression, guidelines recommend that every patient with depression should be assessed for the presence, nature, location, and severity of pain complaints [16].

### Pharmacological treatment

In this epidemiological study, nearly all patients with depression received treatment with antidepressants, which are specified as the first line of treatment in the guidelines (National Institute for Health and Care Excellence (NICE) 2022, American Psychiatric Association (APA) 2010) [17, 18]. According to the guidelines of the APA, patients with mild to moderate major depressive disorder are recommended to be treated initially with an antidepressant, and those with severe major depressive disorder should also receive an antidepressant unless electroconvulsive therapy is planned [17]. The most commonly prescribed medicines in our study were escitalopram and sertraline. Patients were frequently treated concomitantly, with psychiatric pharmacological treatment options being more commonly prescribed than non-psychiatric therapies. The most frequently concomitantly prescribed medicines were anxiolytics, antipsychotics or antiepileptics. The most commonly used antidepressants, escitalopram and sertraline, were administered at recommended doses, whereas duloxetine was slightly below the recommended dose [19–21]. A balanced activity on serotonin and noradrenaline is exhibited by duloxetine at the recommended starting and maintenance dose of 60 mg in the treatment of depression [22].

Statistical analysis and further investigation of treatment patterns among subgroups of included patients with depression based on specific comorbidities were not performed in this study. According to literature data, there is a need to personalize antidepressant treatment in order to enhance treatment outcomes by maximizing the chances of improvement and minimizing the risk of adverse events. The choice of the initial treatment modality should consider several factors such as the symptom profile, presence of co-occurring disorders or psychosocial stressors, the patient’s prior treatment experience, and patient preference [17].

Selective serotonin reuptake inhibitors (SSRI) are considered the first-line treatment for uncomplicated depression or comorbidity with a spectrum of anxiety disorders. The most used SSRI is escitalopram [23]. As per Cipriani A, 2018, escitalopram is considered a favourable SSRI in terms of its efficacy and acceptability balance [24].

In most cases of comorbidity with cardiovascular diseases, SSRIs are considered the first-line antidepressant agents because of their more acceptable safety profile and wider margins of nontoxic levels compared with other antidepressant classes [25]. According to the 2021 guidelines of the European Society of Cardiology (ESC), patients who have coronary heart disease (CHD) and moderate to severe major depression should be evaluated for antidepressant treatment with SSRIs, as this type of treatment can lower the rates of CHD readmission and all-cause mortality [26]. However, there are pharmacological differences among different SSRIs [25]. Sertraline has been suggested to be one of the first-choice antidepressant agents for patients with coronary artery disease (CAD) [25, 27]. According to Stahl, 2017, sertraline has the best documented cardiovascular safety of any antidepressant [28].

In terms of improving pain symptoms, antidepressant treatment has been associated with reductions in pain symptoms among individuals with psychogenic or somatoform pain disorders. For neuropathic pain in general, evidence-based guidelines recommend the use of tricyclic antidepressants (TCAs) or serotonin and noradrenaline reuptake inhibitors (SNRIs) [17]. Given the greater tolerability of SNRI antidepressants, these agents may sometimes be chosen before a TCA for a patient with co-occurring depression and neuropathic pain. Besides, duloxetine is the only antidepressant with official indication for treatment of diabetic peripheral neuropathic pain. Duloxetine as a dual antidepressant reduces pain symptoms by potentiating descending pain inhibitory pathways in the central nervous system. In the USA it is also approved for other indications (fibromyalgia, chronic musculoskeletal pain) [17, 29].

## Conclusion

The study findings corroborate results of earlier research studies conducted some time ago on the high prevalence of psychiatric and non-psychiatric comorbidities in patients with depression. It would be important if future studies could prove the importance of those comorbidities on clinical severity, choice of treatment, and its outcome in patients with depression. Recognizing the comorbidities of depression is crucial as some of these conditions often go unnoticed and are overlooked, which can have serious and unnecessary negative consequences for patients. Therefore, it is imperative to view depression through the lens of comorbidity in order to improve the quality of medical treatment and alleviate the suffering of patients, regardless of their primary diagnosis.
